# A systematic determination of polyphenols constituents and cytotoxic ability in fruit parts of pomegranates derived from five Chinese cultivars

**DOI:** 10.1186/s40064-016-2639-x

**Published:** 2016-06-29

**Authors:** Rui Li, Xiang Gui Chen, Kun Jia, Zhen Ping Liu, Hai Yan Peng

**Affiliations:** Key Laboratory of Food Biotechnology of Sichuan Province, School of Food and Bioengineering, Xihua University, Chengdu, 610039 People’s Republic of China

**Keywords:** Pomegranate, Polyphenols, Chinese cultivars, Quantitative analysis, Cytotoxicity

## Abstract

Plant polyphenols derived from pomegranates are natural health-promoting components, and their bioactivities are well proved. However, the systematic studies of polyphenols constituents and cytotoxic ability in fruit parts of pomegranates derived from different Chinese cultivars have not been studied yet. In this report, a validated and sensitive HPLC–DAD method and fluorescence spectrophotometric method was established for quantitative analysis of four polyphenols and total phenolic content (TPC) in fruit parts of pomegranates (including peels, flesh, seeds, juices and leaves) derived from five Chinese cultivars, respectively. HPLC analysis was performed on the YMC ODS-A C_18_ column with gradient elution of MeOH and 0.1 % TFA. Four polyphenols including gallic acid, ellagic acid, punicalagin A&B and punicalin A&B exhibited satisfactory linearity in the concentration ranges of 20–320, 39–624, 74–1184 and 38–608 μg/mL, respectively. The results demonstrated that the amounts of TPC and four polyphenols in different fruit parts of pomegranates varied significantly. Peels of Sour-YRP possessed the highest content of punicalagin A&B (125.23 mg/g), whereas other three polyphenols exhibited only trace. Among the five Chinese cultivars, Sour-YRP contained the highest content of TPC (688.61 mg/g) and could be considered as the desirable botanical source to obtain polyphenols. It is also discovered that low-maturity pomegranate might possessed much higher TPC than high-maturity pomegranate. The optimized HPLC–DAD method could be used for quality control of different pomegranates by identification and quantification of its main polyphenolic components. Furthermore, the in vitro cytotoxicity of different pomegranates fruit parts to cancer cells was evaluated. We discovered that peels and flesh extract of Sour-YRP significantly inhibited the proliferation of HepG2 and Hela cancer cells lines. The results of this work are promising for further investigation and development of pomegranates as therapeutic agent for the treatment of cancer.

## Background

Pomegranate (*Punica granatum* L.) is an famous ancient fruit which has been extensively cultivated and consumed in many different countries for more than thousands of years (Ismail et al. 2012; Ozgena et al. 2008). Apart from its delicious flavor, the peel, fresh, seeds, juices and leaves of pomegranate also possess a wide range of biological activities due to the high amount of polyphenolic components, including anti-oxidant (Mousavinejada et al. 2009; Zhang et al. 2010), anti-inflammatory (Lee et al. 2008; Romier et al. 2008), anti-microbial (Naz et al. 2007; Voravuthikunchai et al. 2005), anti-cancer (Larrosa et al. 2006; Manasathien et al. 2011; Banerjee et al. 2011) and anti-infective (Gil et al. 2000; Kotwal 2008). The peel, fresh and leaves had already been widely used as Traditional Chinese medicine in China for centuries (Zhou et al. 2008). In addition, Pomegranate was proven to be non-toxic even at high dosages, and exhibit great promise as therapeutic drug to treat various human diseases (Patel et al. 2008; Heber et al. 2007). Polyphenols such as gallic acid, ellagic acid, punicalagin A&B and punicalin A&B are the major chemical component enriched in pomegranate, and pomegranate possessed the highest concentration of punicalagin A&B among the commonly consumed natural fruits (Cuccioloni et al. 2009; Fischer et al. 2011). Previous studies had reported that the major contribution to the anti-oxidant activity of pomegranate is attributed to punicalagin A&B, and other polyphenols also exhibited multiple biological activities such as anti-microbial and anti-infective (Cerda et al. 2003; Lei et al. 2007).

A number of Chinese pomegranate cultivars are cultivated in China, including Sweet Qingpi Pomegranate, Sweet Hongpi Pomegranate, Sour Hongpi Pomegranate, Sour Yunnan Pomegranate and Sweet Tai-mountain Red Pomegranate. The above Chinese pomegranate cultivars represent the most famous pomegranate grown in China and were widely purchased for flesh consumption and industrial production such as concentrated-flesh juices and health-promoting food. However, to the best of our knowledge, the systematic determination of total phenolic and major polyphenols (such as gallic acid, ellagic acid, punicalagin A&B and punicalin A&B), as well as the study of cytotoxicity of different fruit parts of pomegranates derived from Chinese pomegranate cultivars described above remains unclear. The above information is critically important for the quality control and quality assessment of pomegranate from different cultivars.

The aim of this work was to conduct a systematic determination of polyphenols constituents and cytotoxic ability in different fruit parts of pomegranates (including peel, flesh, seeds, juices and leaves) derived from five Chinese cultivars. In the present study, a validated fluorescence spectrophotometric method and HPLC–DAD method was established for quantitative analysis, and 15 batches of pomegranate samples collected from the authentic cultivation areas were determined.

## Experimental

### Plant material

Pomegranate fruits from five distinctive Chinese pomegranate cultivars (*P*. *granatum* L.) were chosen in our research, including Sweet green-peel pomegranate (Sweet-GP; Green-peel), Sweet red-peel pomegranate (Sweet-RP; Red-peel), Sour red-peel pomegranate (Sour-RP; Red-peel), Sour Yunnan Red-peel pomegranate (Sour-YRP; Red-peel) and Sweet Tai-mountain Red-peel pomegranate (Sweet-TRP; Red-peel). Sweet-GP, Sweet-RP and Sour-RP were collected from Huili county of Sichuan province, which is well known as “The hometown of Chinese pomegranate”. Sour-YRP was collected from Mengzi county of Yunnan province, which is also another authentic pomegranate cultivation area in China. Sweet-TRP was collected from Taian city of Shandong province, where possess the pomegranate cultivation history for more than 300 years. All cultivars samples were deposited in school of food and bioengineering, Xihua University.

### Chemicals and reagents

**Fig. 1:**
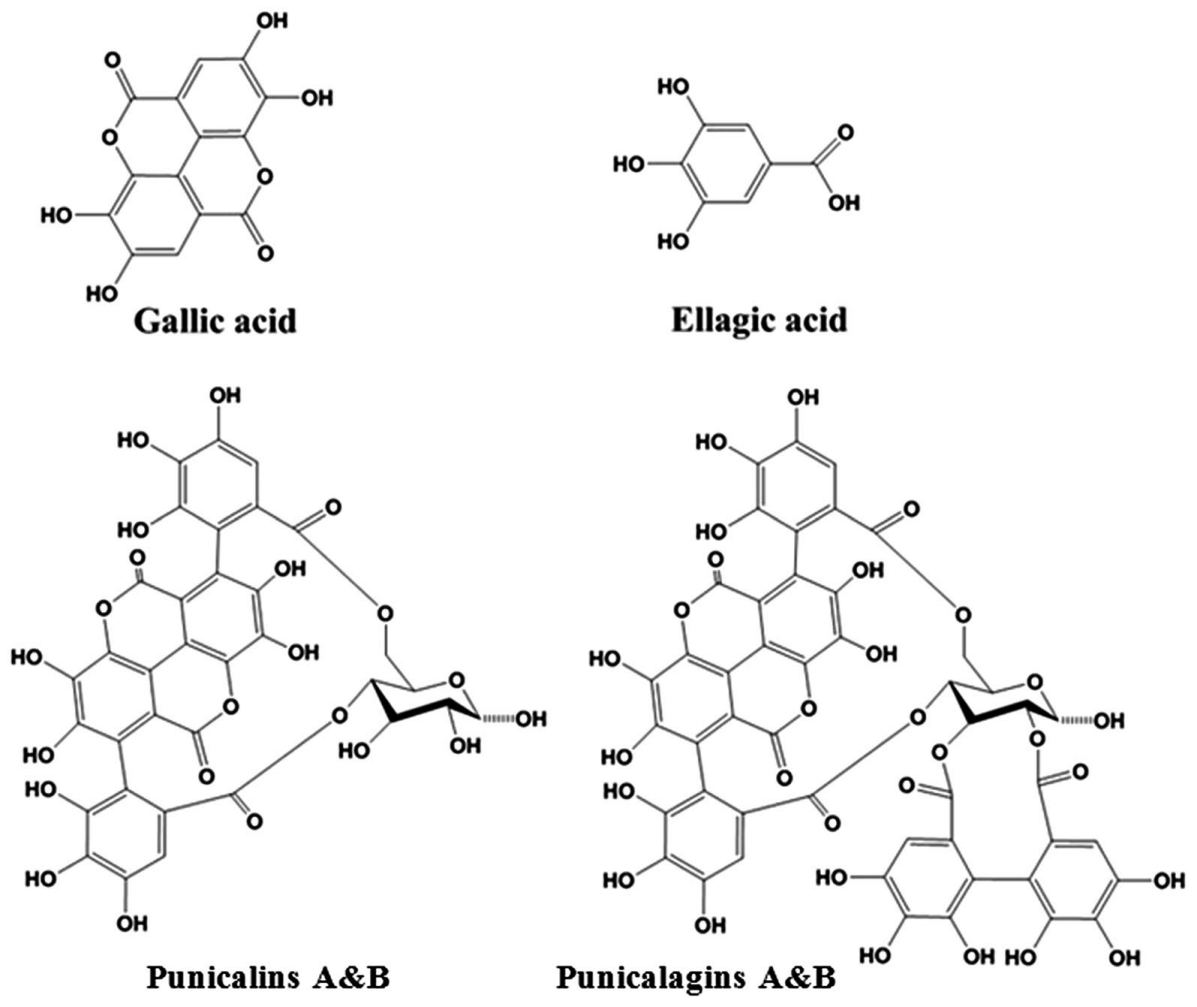
Chemical structures of four polyphenols from pomegranate

All chemicals and solvents were of the HPLC analytical grade. Methanol and trifluoroacetic acid (TFA) were from J.T. Baker (Phillipsburg, NJ, USA). De-ionized water was prepared by a Milli-Q system (Millipore, MA, USA). The reference compounds: gallic acid and ellagic acid were purchased from National Institutes for Food and Drug Control. A mixture of punicalagin A&B (52 % punicalagin A and 48 % punicalagin B) and a mixture of punicalin A&B (52 % punicalin A and 48 % punicalin B) were purchased from Weikeqi-Biotech Co. (Chengdu, China). The Chemical structures of four polyphenols were exhibited in Fig. 1.

### Instrumentation and conditions

Spectrophotometric assays were performed on a Varian Cary 1E spectrophotometric system (Palo Alto, CA, USA). HPLC chromatographic analysis was performed on an Agilent series 1100 system (Santa Clara, CA, USA) including a quaternary pump, a variable wavelength detector, an autosampler, and a column compartment was used. The separation was performed on a YMC ODS-A C_18_ reversed column (250 mm × 4.6 mm i.d., 5 μm) equipped with an Agilent Zorbax SB-C_18_ guard column (12.5 mm × 4.6 mm i.d., 5 μm). The mobile phase consisted of MeOH (A) and 0.1 % TFA in water (B) using a gradient program of 2–13 % (A) in 0–10 min, 13 % (A) in 10–20 min, 13–40 % (A) in 20–25 min, 40 % (A) in 25–45 min. The mobile phase flow rate was 1.0 mL/min and the column temperature was set at 35 °C. The DAD detector was monitored at 254 nm for quantitative analysis of gallic acid and 377 nm for ellagic acid, punicalagin A&B and punicalin A&B, respectively.

### Determination of total phenolic content (TPC) by fluorescence method

The determination of total phenolic content (TPC) was completed by using fluorescence spectrophotometric system according to Folin–Ciocalteau method (Li et al. 2009). The gallic acid solution was used as standard and the result was expressed as milligrams of TPC equivalents *per* grams of air-dried powder of different fruit parts. The pomegranate of different cultivars were collected, classified and pretreated. The fresh pomegranate juices of each cultivar were obtained by manual juicer (avoid broking the seeds) and filtered through a 0.45 μm membrane and an aliquot of 2 mL was diluted to 25 mL with de-ionized water in a volumetric flask for analysis. The pomegranate peel, flesh, seeds and leaf samples were air-dried and pulverized into a fine powder. An aliquot of 1.0 g of each powder was extracted twice with 25 mL of 70 % acetone in an ultrasonic bath (40 kHz, 300 W) for 60 min at 35 °C. After centrifugation at 8000 rpm for 5 min, all the supernatant was combined and organic layer was evaporated (35 °C) by a rotary evaporator. The residue was dissolved and diluted with de-ionized water to 250 mL. Finally, an aliquot of 2 mL solution was diluted to 100 mL in a volumetric flask and used to spectrophotometric analysis.

### Determination of polyphenols by HPLC–DAD method

#### Sample preparation

The fresh pomegranate were collected, pretreated and classified according to different cultivars. The pomegranate peel, flesh, seeds and leaves samples of each cultivar were air-dried and pulverized into a fine powder. An aliquot of 2.0 g of each powder was extracted twice with 100 mL of mixed solution (consist of methanol–ethanol–aceton–water, 25 mL of each) in an ultrasonic bath (40 kHz, 300 W) for 60 min at 35 °C. After centrifugation at 12,000*g* for 5 min, the upper organic layer was transferred to another tube. The remainder water layer was extracted with 75 mL ethyl acetate for 3 times, then all the upper organic layer was combined and evaporated to dryness. The residue was reconstituted in 50 mL methanol followed by ultrasonication for 1 min. The solution was filtered through a 0.45 μm membrane and a 50 μL aliquot of supernatants was injected into the HPLC system for analysis. For the pomegranate juices sample processing, an aliquot of 50 mL of the juices was extracted 3 times with 50 mL ethyl acetate, and followed by the same procedure as described previously. Then the methanol solution was evaporated to dryness, dissolved with an appropriate quantity of methanol, and was finally diluted to 10 mL in a volumetric flask. The solution was filtered through a 0.45 μm membrane and a 50 μL aliquot was used for HPLC analysis.

#### Preparation of standard solutions

Gallic acid, ellagic acid, punicalagin A&B and punicalin A&B were accurately weighed and dissolved in methanol solutions, and the concentration of four polyphenols was 400, 390, 380 and 370 μg/mL, respectively. An aliquot of 50, 100, 100 and 200 μL of each analytes was mixed and diluted to 1 mL in a volumetric flask, and then stored at 4 °C before HPLC analysis. The final concentration of four compounds in stock solution was 20 (gallic acid), 39 (ellagic acid), 38 (19.76 μg/mL, punicalagin A; 18.24 μg/mL, punicalagin B) and 74 μg/mL (38.48 μg/mL, punicalin A; 35.52 μg/mL, punicalin B), respectively.

### MTT assay

The cells were cultivated in CO_2_ incubators with 5 % CO_2_ at 37 °C in DEME medium. Once the cells reached 90 % confluence, the cells were inoculated in 96 wells plates with a seeding density of 45,000 cells/well and kept for 24 h of incubation. Further the cells were incubated with 100 μL of sample solutions in media for 48 h, then 20 μL of MTT solution (5 mg/mL in PBS) was added to each well. After incubated for additional 4 h, the medium was removed and 150 μL of DMSO (dimethyl sulfoxide) was added to each well. The cells were agitated with an orbital shaker for 10 min, then OD values of the cells were measured using a enzyme-labeling instrument. The formazan crystals produced had an absorption maximum of 490 nm, so the cell viability can be quantified using the OD values at 490 nm. The cell viability were calculated by the following formula: cell viability (%) = absorbance of the cells treated with the samples/absorbance of the untreated cells × 100 %. A control group (medium without samples, 100 % viability) and a blank group (without cells, 0 % viability) were also included. Data were presented as means ± standard deviations. All the experiments were done in triplicates.

## Results and discussion

### Method validation (fluorescence method)

The proposed fluorescence spectrophotometric method for quantitative analysis of total phenolic content (TPC) was validated in terms of linearity, precision, repeatability and recovery.

#### Linearity

Working solutions of six concentrations containing gallic acid were analyzed in triplicate to obtain the calibration curve. The calibration curves were established by plotting peak areas versus the concentration of analyte and in the regression equation *y* = a*x* + b, *x* refers to the concentration of gallic acid (mg/mL), *y* the peak area of absorbance, and *r* the correlation coefficient. The final regression equation was *y* = 4.1097*x* + 0.0194 (*r* = 0.9995), and calibration curves of gallic acid showed good linearity in relatively wide dynamic ranges from 0.001 to 0.040 mg/mL.

#### Precision

Intra- and inter-day variations were used to determine the precision. The gallic acid solution were determined for six times within the same day for intra-day test, while for inter-day test, the samples were examined twice per day in three consecutive days. The method exhibited good precision with the RSD of analyte of intra-day and inter-day was <1.23 %.

#### Repeatability

To confirm the repeatability of analyte, six replicates of different sample solutions including peel, flesh, seeds, juices and leaves were prepared and analyzed and the calculated results were 0.32, 2.47, 1.10, 0.83 and 1.64 % respectively, revealed good reproducibility of the method.

#### Recovery

Recovery test was used to evaluate the accuracy of the method. Gallic acid was added to different sample solutions including peel, flesh, seeds, juices and leaves, and then extracted and analyzed as described for normal samples. The recoveries were determined by the formula: recovery (%) = (amount found–original amount)/amount spiked × 100 %, respectively. The present method exhibited satisfactory accuracy with the recovery of each sample solution ranging from 91.05 to 98.21 %.

### Method validation (HPLC method)

#### Linearity, the limits of detection and quantification

In the linearity test, working solutions of six concentrations containing gallic acid, ellagic acid, punicalagin A, punicalagin B, punicalin A and punicalin B were analyzed in triplicate. In the regression equation *y* = a*x* + b, *x* refers to the concentration of all analytes (mg/mL), *y* the peak area, and *r* the correlation coefficient. The limit of detection (LOD) and limit of quantification (LOQ) were calculated by injecting a series of standard solutions until the signal-to-noise (S/N) ratio was 3 for LOD and 10 for LOQ, respectively. Calibration curves of the all analytes exhibited good linearity in wide dynamic ranges (calibration equation, LODs, and LOQs are listed in Table 1).

**Table 1:** Regression equations, LOD and LOQ of all analytes

Analytes	Regression equation	Dynamic range (μg/mL)	*r*	LOD (ng/mL)	LOQ (ng/mL)
Gallic acid	*y* = 2353.15*x* − 17496	20–320	0.9997	20.5	64.5
Ellagic acid	*y* = 1112.91*x* − 18658.51	39–624	0.9994	18.8	52.0
Punicalagin A	*y* = 413.95*x* − 12127.42	38.5–615.7	0.9978	12.4	50.2
Punicalagin B	*y* = 382.11*x* − 11194.54	35.5–568.3	0.9985	11.5	46.3
Punicalin A	*y* = 880.44*x* − 23405.09	19.8–316.2	0.9971	10.5	46.5
Punicalin B	*y* = 812.71*x* − 21604.70	18.2–291.8	0.9965	9.7	42.9

#### Precision, reproducibility and stability

Intra- and inter-day variations were used to determine the precision. For intra-day test, the samples of each analyte were analyzed for six times within the same day, and for inter-day test, the samples were examined thrice per day in two consecutive days. Reproducibility was evaluated by extracting and analyzing six replicates of the same sample according to the established method. For stability test, the sample solution was analyzed every 6 h for 48 h at room temperature. The intra-day and inter-day RSD of all analytes ranged from 0.92 to 2.30 %, indicating good precision for quantitative analysis. The method exhibited good reproducibility with RSD <2.92 % and all analytes were found to have no significant changes within 48 h’ storage period with RSD <2.98 %.

#### Accuracy

Recovery test was used to evaluate the accuracy of this method. Accurate amount of mixed analytes were added to different sample solutions including peel, flesh, seeds, juices and leaves, and then extracted and analyzed as described for normal samples. The average recoveries were calculated by the formula: recovery (%) = (amount found − original amount)/amount spiked × 100 %. The present quantitative method demonstrated suitable accuracy with the overall recoveries of all analytes ranging from 94.3 to 100.07 % in different samples (results are given in Table 2**)**.

**Table 2:** Recoveries of gallic acid, ellagic acid, punicalagin and punicalin in different sample solutions (n = 3)

Analytes	Peel (%)	Flesh (%)	Seeds (%)	Juices (%)	Leaves (%)
Gallic acid					
Recovery	96.22	98.80	100.06	97.05	97.37
RSD	2.75	2.42	2.64	2.05	2.19
Ellagic acid					
Recovery	99.84	95.81	92.37	98.14	97.52
RSD	1.82	1.90	2.64	2.10	2.43
Punicalagin A					
Recovery	98.00	99.47	96.55	95.30	96.50
RSD	2.11	2.37	2.58	2.03	2.38
Punicalagin B					
Recovery	96.47	97.67	97.25	97.30	97.51
RSD	1.61	2.87	1.58	2.74	2.68
Punicalin A					
Recovery	95.35	94.30	96.49	96.21	97.92
RSD	2.28	1.92	2.41	1.97	1.82
Punicalin B					
Recovery	97.42	96.28	95.78	97.38	98.26
RSD	2.57	2.26	2.63	2.37	2.02

### Determination of total phenolic content (TPC)

#### The comparison of TPC among five Chinese pomegranate cultivars

The established fluorescence spectrophotometric quantitative method was used to determine total phenolic content (TPC) in five Chinese pomegranate cultivars and their corresponding different fruit parts. Three batches of each Chinese pomegranate cultivars samples were collected and analyzed. The data of TPC values are shown in Table 3. Among all the five Chinese pomegranate cultivars samples, Sour-YRP contained the highest amount of TPC with a total amount of 688.61 mg/g in all (including peel, flesh, seeds, juices and leaves), and Sweet-TRP also contained relative high amount of TPC for 602.98 mg/g. Another three Chinese pomegranate cultivars: Sweet-GP, Sweet-RP and Sour-RP possess relative low amount of TPC for 581.69, 522.41 and 557.98 mg/g, respectively. As shown in Table 3, we discovered that the amount of TPC exhibited significant difference in five Chinese pomegranate cultivars, indicating the quality diversity among different cultivation regions. The Sour-YRP cultivated in Mengzi county of Yunnan province and Sweet-TRP cultivated in Taian city of Shandong province contained much higher TPC than Sweet-GP, Sweet-RP and Sour-RP cultivated in Huili county of Sichuan province. The three different cultivars from Huili county possessed similar amount of TPC, indicating the local climate and environment might be critical to their total phenolic content. The above results could be useful for quality assessment of pomegranates from different regions.

**Table 3:** Contents (mg/g d.w) of four polyphenols and total phenolic content (TPC) in different fruit parts of pomegranates derived from five Chinese cultivars (n = 3)

Medicinal parts	Chinese cultivars	Cultivation regions	Gallic acid	Ellagic acid	Punicalagin A&B	Punicalin A&B	TPC
Peel	Sweet-GP	Huili, Sichuan	0.27 ± 0.03	4.08 ± 0.42	103.61 ± 7.36	1.89 ± 0.04	264.58 ± 8.74
Flesh			0.07 ± 0.01	1.85 ± 0.16	44.77 ± 2.37	0.71 ± 0.01	217.14 ± 16.80
Seeds			0.03 ± 0.01	0.05 ± 0.01	nd	nd	9.04 ± 1.23
Juices			0.06 ± 0.01	0.02 ± 0.004	0.34 ± 0.02	0.01 ± 0.004	8.62 ± 1.05
Leaves			0.03 ± 0.002	7.02 ± 0.15	1.67 ± 0.11	0.15 ± 0.005	82.31 ± 2.26
Peel	Sweet-RP	Huili, Sichuan	0.41 ± 0.06	7.07 ± 0.46	61.75 ± 4.69	3.91 ± 0.45	231.36 ± 4.40
Flesh			0.07 ± 0.01	1.78 ± 0.26	42.38 ± 2.42	0.59 ± 0.08	203.20 ± 12.68
Seeds			0.02 ± 0.002	0.03 ± 0.003	nd	nd	6.17 ± 0.60
Juices			0.09 ± 0.01	0.02 ± 0.003	0.01 ± 0.04	0.01 ± 0.005	6.12 ± 0.91
Leaves			0.02 ± 0.002	3.61 ± 0.76	0.77 ± 0.06	0.09 ± 0.01	75.56 ± 4.39
Peel	Sour-RP	Huili, Sichuan	0.26 ± 0.02	4.09 ± 0.43	94.11 ± 1.84	1.68 ± 0.22	255.31 ± 6.42
Flesh			0.07 ± 0.01	2.11 ± 0.11	50.6 ± 1.49	1.24 ± 0.12	208.39 ± 6.31
Seeds			0.02 ± 0.004	0.03 ± 0.003	nd	nd	8.31 ± 0.83
Juices			0.14 ± 0.03	0.02 ± 0.003	0.61 ± 0.04	0.01 ± 0.002	10.36 ± 1.12
Leaves			0.02 ± 0.002	3.56 ± 0.52	0.79 ± 0.03	0.09 ± 0.012	75.61 ± 3.62
Peel	Sour-YRP	Mengzi, Yunan	0.18 ± 0.02	3.37 ± 0.39	125.23 ± 13.10	2.72 ± 0.23	302.43 ± 9.54
Flesh			0.11 ± 0.01	0.39 ± 0.05	62.68 ± 2.74	1.01 ± 0.09	272.10 ± 9.98
Seeds			0.02 ± 0.002	0.02 ± 0.006	nd	nd	12.44 ± 0.96
Juices			0.05 ± 0.007	0.02 ± 0.001	0.70 ± 0.02	0.01 ± 0.004	13.31 ± 0.87
Leaves			0.04 ± 0.002	4.96 ± 0.03	2.21 ± 0.23	0.15 ± 0.03	88.33 ± 1.92
Peel	Sweet-TRP	Taian, Shandong	0.19 ± 0.03	2.68 ± 0.12	71.10 ± 5.03	2.01 ± 0.05	279.76 ± 13.32
Flesh			0.08 ± 0.01	0.54 ± 0.08	47.63 ± 1.13	0.78 ± 0.14	222.62 ± 7.72
Seeds			0.02 ± 0.003	0.03 ± 0.003	nd	nd	8.59 ± 1.04
Juices			0.04 ± 0.006	0.02 ± 0.002	0.22 ± 0.03	0.01 ± 0.003	12.91 ± 0.87
Leaves			0.02 ± 0.006	4.02 ± 0.21	1.33 ± 0.079	0.12 ± 0.015	79.10 ± 2.74

*nd* not detected, *d.w* dry weight

#### The comparison of TPC among different fruit parts

The TPC amount of peel, flesh, seeds, juices and leaves of the pomegranate also exhibited great difference among five cultivars. As shown in Table 3, peel and flesh of the pomegranate contained much higher TPC amount than seeds, juices and leaves, and the peel together with flesh accounted for more than 83 % of total TPC amount of an entire pomegranate. We found the same result among five Chinese pomegranate cultivars, indicating that polyphenols were more enriched in peel and flesh, while were scarcity in seeds and juices. However, the peel of pomegranate was normally discarded after acquiring the flesh and juices, it is suggested that this fruit part should be recovered and utilized in view of its high levels of polyphenols amounts.

#### The comparison of TPC among different maturity (low, medium, high)

**Fig. 2:**
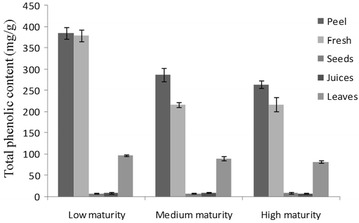
The quantitative comparison of total phenolic content (TPC) in Sweet-GP with different maturity (low, medium, high)

In our research, we also discovered that TPC amount of pomegranate was varied according to their different maturities. Herein we take Sweet-GP as an example. The pomegranate fruits were classified into three maturities, including low (Unripe), medium (half-Ripe) and high (Ripe) were collected and analyzed by the established method and the results were shown in Fig. 2. Interestingly, the low-maturity pomegranate contained the highest amount of TPC with a total amount of 878.22 mg/g, whereas high-maturity pomegranate only contained the lowest amount of 581.89 mg/g. Our study indicated that total phenolic content of pomegranate were reduced significantly during the growing period from low-maturity to high-maturity. Moreover, previous reports had demonstrated that the multiple biological activities of pomegranate were mainly attributed to its polyphenolic components, especially for polyphenols in peel (Li et al. 2006). Thus the high-content polyphenols in low-maturity pomegranate could be critically important for its potential health application.

Taken together, despite the five Chinese pomegranate cultivars used in our research are representative cultivars in China, their amounts of TPC were significantly different. Sour-YRP could be considered as the desirable botanical source of polyphenols. In addition, it was also suggested that TPC were higher in low-maturity pomegranate than in high-maturity pomegranate, and the peel and flesh of pomegranate could be more promising as a potential anti-oxidant and anti-inflammatory drug than the other fruit parts due to the high polyphenolics amount.

### Determination of four polyphenols

**Fig. 3:**
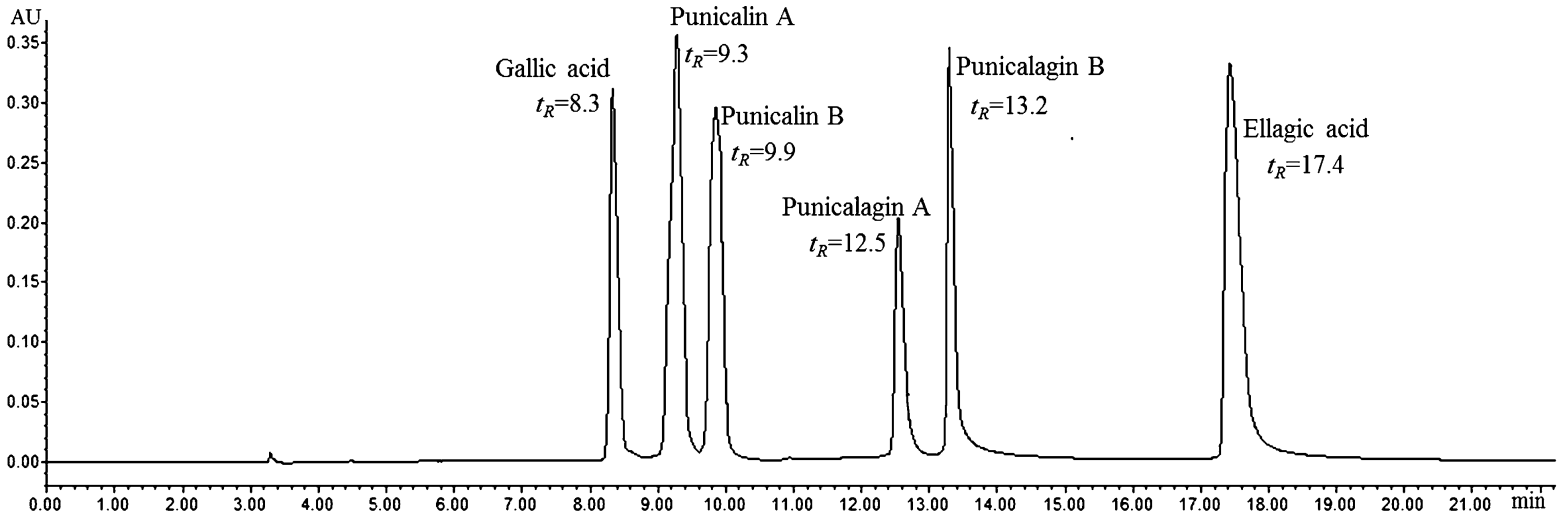
HPLC chromatograms of polyphenols from pomegranate (detected wavelength was 377 nm)

The established HPLC–DAD quantitative method was applied to determine contents of four polyphenols including gallic acid, ellagic acid, punicalagin A&B (C_punicalagin A&B_ = C_punicalagin A_ + C_punicalagin B_) and punicalin A&B (C_punicalin A&B_ = C_punicalin A_ + C_punicalin B_) in different fruit parts. As shown in Fig. 3, six analytes including two pairs of isomers (punicalagin A and punicalagin B; punicalin A and punicalin B) were successfully separated using our HPLC condition. A total of 15 batches of different Chinese pomegranate cultivars samples were collected and analyzed (Data were listed in Table 3). The comparison of calculated contents of gallic acid, ellagic acid, punicalagin A&B and punicalin A&B in peel, flesh, seeds, juices and leaves from five Chinese pomegranate cultivars were examined. It is discovered that the contents of four polyphenols were remarkably different in peel, flesh, seeds, juices and leaves of five Chinese pomegranate cultivars. Among them, peel and flesh of Sour-YRP possessed the highest content of four polyphenols, whereas that of Sweet-RP contained the lowest (see Table 3), which was in accordance with the result found in quantitative analysis of TPC. Moreover, it is also found that the content of punicalagin A&B in peel of Sour-YRP (125.23 mg/g) was much higher than other polyphenols in five cultivars, whereas gallic acid, ellagic acid and punicalin A&B possessed only trace in different parts of pomegranate with the content ranged from 0.02 (seeds) to 0.41 (peel, Sweet-RP), 0.02 (juices) to 4.08 (peel, Sweet-RP) and 0.01 (juices) to 3.91 mg/g (peel, Sweet-RP), respectively. Interestingly, punicalagin A&B and punicalin A&B were not detected in seeds of five cultivars, considering that the TPC, as well as gallic acid, ellagic acid was also trace in this part, it appeared that the nutritional value of seeds might be limited.

### Evaluation of cytotoxic activity to tumor cells

**Fig. 4:**
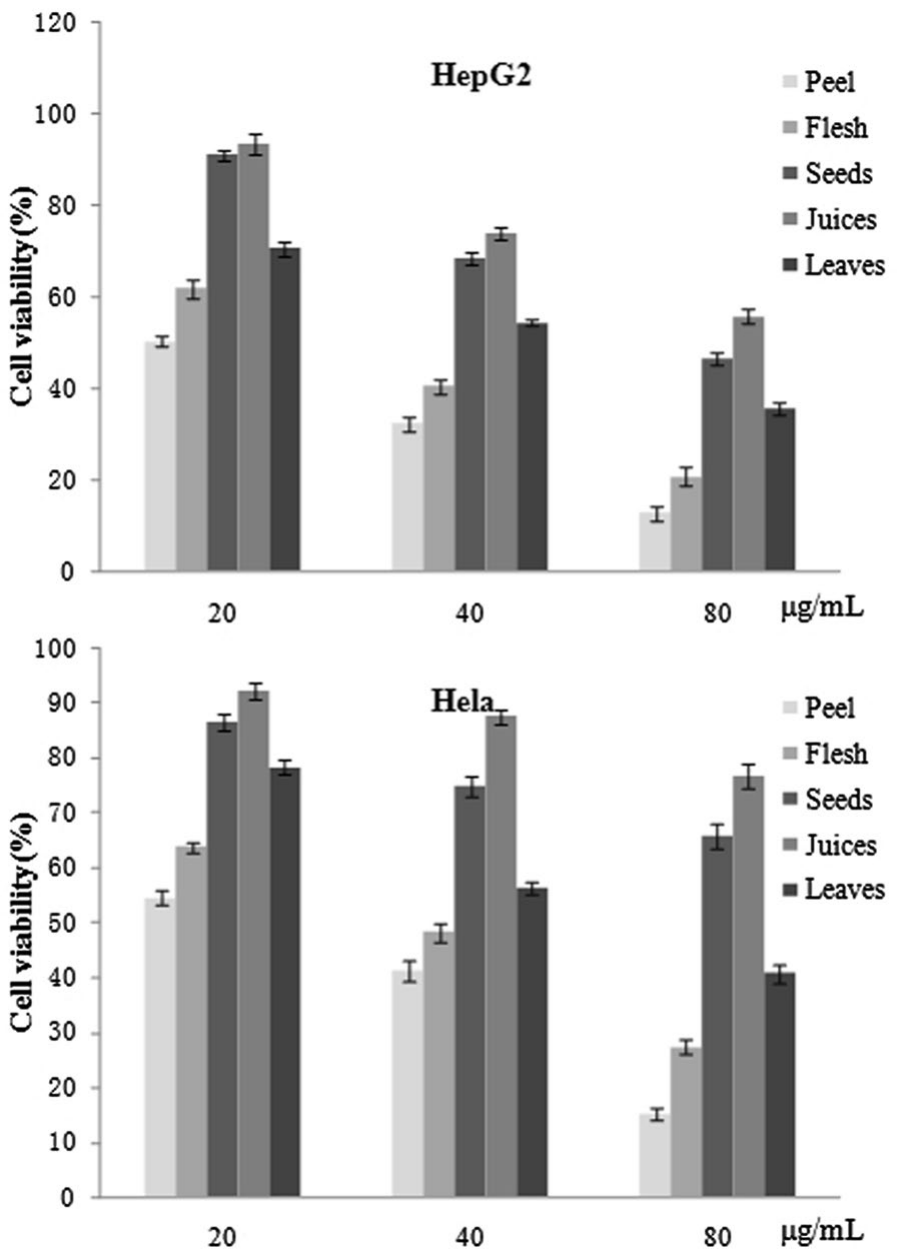
The cell viability of HepG2 and Hela tumor cells treated with different concentrations of extractions of peel, flesh, seeds, juices and leaves (a Student’s *t* test was used to determine statistical significance, *P* < 0.05)

In this paper, the inhibitory effect of different fruit parts of Sour-YRP (peel, flesh, seeds, juices and leaves) were tested by MTT assay. The results demonstrated that the inhibitory effect to HepG2 and Hela cells of fruit parts were different (see Fig. 4). Interestingly, the peel and flesh of pomegranate exhibited significantly potent cytotoxicity to both HepG2 and Hela cells in low, medium and high concentrations compared to the other fruit parts. The best inhibitory effect was observed at the highest concentration of peel sample (80 μg/mL); the HepG2 and CT-26 cell viability was decreased to 12.7 ± 2.5 and 15.2 ± 2.7 %, respectively. Furthermore, treatment of tumor cells with seeds and juices resulted in a limited cell growth inhibition than peel and flesh. According to our results that peel and flesh accounted for more than 83 % of total TPC amount of an entire pomegranate. We speculate that the cytotoxicity of the fruit parts was related to their TPC amount. Previous study (Jalila et al. 2013) have reportted that the methanolic extract of pomegranate leaves exhibited promising cytotoxic activity with IC50 values of 31 ± 1.02 μg/mL, which also indicated that the high amount of total phenolics in pomegranate extract predominantly contributed to the cytotoxic activity. Taken together, these data suggest that the peel of pomegranate is a promising therapeutic agent for the treatment of tumor.

## Conclusion

In summary, the systematic analysis of polyphenols constituents and cytotoxic ability in different fruit parts of pomegranates were established. The pomegranates samples derived from five Chinese cultivars were used in our study. With the help of fluorescence spectrophotometric method and HPLC–DAD method, the systematic determination of total phenolic content (TPC) and four polyphenols in different fruit parts of pomegranates were characterized and simultaneously determined. Our results demonstrated that the content of TPC and four polyphenols varied significantly in all the samples. Punicalagin A&B exhibited the highest amount in peel of Sour-YRP, whereas other three polyphenols exhibited only trace in pomegranate. Among different fruit parts of five cultivars, peel and flesh of Sour-YRP could be considered as the suitable botanical source for extracting the polyphenol constituents. We also discovered that the low-maturity pomegranate possessed much higher TPC than the high-maturity pomegranate, and this should be taken into account during extracting the total polyphenols. Furthermore, the efficacy of different fruit parts of pomegranates as a potential chemotherapeutic agent was evaluated using the MTT assay to measure inhibition of cell growth in HepG2 and Hela cancer cells. Treatment of these cell lines with the extract of pomegranates fruit parts resulted in significant inhibitory action on the growth of HepG2 liver cancer cells and Hela carcinoma cells. The peels and flesh extract of Sour-YRP possessed significant cytotoxicity, which indicated these fruit parts of pomegranate could be more valuable than juices for health-promoting using. To the best of our knowledge, this is the first systematic research to study polyphenols constituents and cytotoxic ability in different fruit parts of pomegranates derived from five Chinese cultivars. Currently, we are studying the cellular mechanism of cytotoxicity of polyphenols constituents from pomegranates. The results of the current study have potential applications for further investigation and development of pomegranates as therapeutic agent for the treatment of cancer, and the optimized analytical method developed can be used for comprehensive quality control of polyphenols constituents of pomegranates.
